# Cannabinoid receptor 2 deletion deteriorates myocardial infarction through the down-regulation of AMPK-mTOR-p70S6K signaling-mediated autophagy

**DOI:** 10.1042/BSR20180650

**Published:** 2019-04-26

**Authors:** Yao Hu, Yu Tao, Jing Hu

**Affiliations:** 1 Cardiovascular Medicine Department, Jiangxi Provincial People’s Hospital, 152 Aiguo Road, Nanchang, Jiangxi, China; 2 Cardiovascular Medicine Department, The First Hospital Of Nanchang, 128 Xiangshan North Road, Nanchang, Jiangxi, China

**Keywords:** Autophagy, Cannabinoid receptor 2, Myocardial infarction, rapamycin

## Abstract

Cannabinoid receptor 2 (CB2R) has been reported to play an important role in the regulation of pathogenesis and progression of myocardial infarction (MI). Here we tried to investigate its potential mechanisms. The ratio of infarct size in heart issue was detected by TTC staining, and cardiac functions were calculated according to echocardiographic evaluation. Cell viability in cardiomyocytes was investigated by Cell Counting Kit-8 (CCK-8) and lactate dehydrogenase (LDH) release assays. Western blot was used to detect autophagy-related proteins including Beclin-1, LC3, p62, adenosine 5′-monophosphate (AMP)-activated protein kinase (AMPK)-mammalian target of rapamycin rabbit (mTOR)-p70 ribosomal protein S6 kinase (p70S6K) signaling-related proteins including AMPK, mTOR, p70S6K, and their phosphorylation formation. Rapamycin was used for the induction of autophagy. Cleaved caspase-3 and Bax were detected for analyzing apoptosis. TEM was used for the detection of autophagosomes. We found that CB2R deletion (CB2R KO) largely deteriorated the severity of MI and the cardiac function as well as cell viability of cardiomyocytes. Knocking out CB2R decreased the level of autophagy in heart issues from MI mice as well as cardiomyocytes under oxygen-glucose deprivation (OGD). Furthermore, CB2R dysfunction significantly attenuated the cardiac protective effects of rapamycin both *in vivo* and *in vitro*. Finally, we found that CB2R-mediated autophagy was induced by AMPK-mTOR-p70S6K signaling pathway. Our current study demonstrated for the first time that CB2R deletion led to a detrimental effect of MI through the dysfunction of AMPK-mTOR-p70S6K signaling pathway, which might provide a novel insight in the treatment of MI.

## Introduction

Myocardial infarction (MI), also known as heart attack, leads to a huge mortality worldwide and causes great damage to people’s health. The population of MI patients is huge, with about 15.9 million MI patients all over the world [1]. It is widely acknowledged that the fundamental factor of MI lies in the damage of heart muscle, thus leading to the infarction of cardiomyocytes, change of blood flow in part of the heart, and deterioration of cardiac function. The major pathogenic factors include atherosclerosis in coronary artery, obesity, high blood pressure, and cholesterol [2,3]. However, the specific mechanisms of MI are not fully elucidated and more effective therapies for MI are demanded.

The endocannabinoid system has been reported to be vital in the regulation of the pathogenesis and progression of diseases [4–6]. In MI, it was shown that changes in endocannabinoid gradients due to altered tissue levels contributed to the improvement of cardiac healing and function due to regulation of myeloid cell recruitment [4]. Cannabinoid receptor 2 (CB2R), together with CB1R, belongs to the family of cannabinoid receptors, which are components of the endocannabinoid system [7]. CB2R is a typical type of G-protein coupled receptor with seven transmembrane domains. Unlike CB1R which mainly located in neurons in charge of neurotransmitter release [8], CB2R is widely spread in peripheral cells and tissues including the immune cells, liver, heart, and brain [9–12]. As a result, CB2R has been reported to regulate the pathogenesis and progression of various kinds of inflammation- and immune-related diseases, such as atherosclerosis and multiple sclerosis [13–18]. In MI, CB2R was demonstrated to produce alleviative effects [19–21]. However, the underlying mechanisms remain unclarified.

Autophagy is an important metabolic mechanism in organisms, functioning in degrading, and recycling long-lived proteins and dysfunctional organelles [22]. Autophagy is involved in the regulation of cerebral-cardiovascular diseases, including atherosclerosis, MI, ischemic stroke, and multiple sclerosis [23–25]. In MI, autophagy has been reported to produce an effect of cardiac protection, thus protecting against the pathogenesis and progression of MI [26]. As a result, properly inducing autophagy might serve as a potential pathway in the treatment of MI.

Here, we raise a hypothesis that activating CB2R protects against MI through the induction of autophagy. We aim to explore new mechanisms of MI and develop novel therapeutic strategy against MI.

## Materials and methods

### Animal care and use

CB2R knockout (KO) and the wild-type (WT) mice on C57BL/6 background (8–10 weeks old, male) were purchased from Jackson Laboratory (Bar Harbor, MA, U.S.A.) (B6.129P2-Cnr2tm1Dgen/J, Stock Number: 005786). Mice were housed in cages at controlled environment (21 ± 4°C, 12 h light/dark) with an ambient humidity of 50–80%. Mice were treated with unlimited access to water and standard rodent diet. All experiments were approved and conducted in accordance with the guidelines of Jiangxi Provincial People’s Hospital.

### MI mice model creation and treatment

MI surgery was conduced on 8- to 9-week-old mice by permanently occluding the left anterior descending coronary artery as described previously [27,28]. In brief, mice were anesthetized with isoflurane (5%) using a ventilation equipment. The heart was exposed through a lateral thoracotomy with a 4–5 intercostal incision. An 8–0 prolene suture was used to ligate the left main coronary artery at 1–2 mm below the ostium. Paleness around and below the ligation point indicated success of surgery. The chest cavity was closed with 7–0 nylon sutures and placed in a warming pad until recovery from anesthesia and surgery.

### Echocardiographic examination

A 2-D guided M-mode echocardiography in a Vevo 2100 system (Vevo 2100, Visual Sonics) was used for the detection of cardiac function as described previously [28]. In brief, mice were anesthetized with isoflurane (5%) using a ventilation equipment and then subjected to echocardiographic examination. Left ventricular end-systolic diameter (LVESD) and left ventricular end-diastolic diameter (LVEDD) were obtained from at least three separate cardiac cycles. Ejection fraction (EF)(%) and fractional shortening (FS)(%) were calculated according to the equations listed as follows: LVESD = 7.0 × LVESD^3^/(2.4 + LVESD); LVESD = 7.0 × LVEDD^3^/(2.4 + LVEDD); EF (%) = (LVEDV−LVESV)/LVESV × 100%; EF (%) = (LVEDD−LVESD)/LVESD × 100%. Those measurements with echocardiography were more accurate indicators of MI size than the more widely used dP/dt by invasive hemodynamics according to a previous report [29].

### TTC staining

TTC staining was conducted for the assessment of infarct size in heart issue as described previously [30]. In brief, at 24-h postinfarction, mice were anesthetized with isoflurane (5%) and heart issues were quickly isolated from mice. After washed with saline, the heart issues were frozen for 10 min and cut into five transverse slices from apex to base of equal thickness (1–1.5 mm). The slices were then stained in 1% TTC, incubated in 37°C for 10 min, and fixed in 4% (w/v) paraformaldehyde for 30 min. The infarct size was assessed using ImageJ software.

### Primary cardiomyocytes culture and treatment

Primary ventricular cardiomyocytes were isolated from neonatal WT or CB2R KO mice as described previously [28]. In brief, the heart issues were harvested and dissociated with 0.05% trypsin (Gibco, Grand Island, NY, U.S.A.) and 0.08% type II collagenase (Gibco, U.S.A.). Cells were resuspended in (DMEM; Gibco) with 10% fetal bovine serum (FBS) and 0.1% penicillin–streptomycin and plated onto a 25-cm^2^ cell culture flask for 90 min at 37°C in a 5% CO_2_ incubator. Unadherent cells were plated onto six-well (1 × 10^6^ cells/well) or 96-well (1 × 10^5^ cells/well) plates and cultured at 37°C in a 5% CO_2_ incubator. For cell experiments, the condition of oxygen-glucose deprivation (OGD), in which primary cardiomyocytes were cultured in glucose-free Earle’s balanced salt solution with 1% O_2_ at 37°C, was used for the creation of *in vitro* model of MI. For the induction of autophagy process, rapamycin (1 μg/l, Selleckchem, Houston, TX, U.S.A.) was administrated for the treatment of cardiomyocytes.

### Cell viability

Cell viability was measured by Cell Counting Kit-8 (CCK-8; Dojindo, Kamimashiki-gun Kumamoto, Japan) according to the manufacturer’s protocol. In brief, primary cardiomyocytes were seeded in 96-well plates in the density of 1 × 10^5^ cells/well. After 6-h OGD, 10 μl CCK-8 reagent was added to each well for 1-h additional cultivation. Absorbance was assayed with a microplate reader (Tecan Group Ltd., Männedorf, Switzerland) at the wavelength of 450 nm for the analysis of cell viability.

### LDH release

LDH release assay (Dojindo, Kamimashiki-gun Kumamoto, Japan) was used for the detection of cell membrane integrity following the manufacturer’s protocol. In brief, primary cardiomyocytes were seeded in 96-well plates in the density of 1 × 10^5^ cells/well. After 6-h OGD, LDH Working Solution was added to each well for the incubation of additional 30 min at 37°C. After stop solution was added, absorbance was assayed with a microplate reader (Tecan Group Ltd., Switzerland) at the wavelength of 490 nm.

### Western blot

Infarcted and normal heart issues as well as primary cardiomyocytes with or without exposure to OGD were lysed in lysis buffer. Bicinchoninic acid method (Thermo Scientific, Pittsburgh, PA, U.S.A.) was conducted for the measurement of total protein concentration. Samples were loaded in 6 or 15% Tris/Gly gels, and transferred on NC membranes through SDS-PAGE (Millipore, Billerica, MA, U.S.A.). Blotting was conducted using the rabbit anti-Beclin-1 monoclonal antibody (1:1000; Cell Signaling Technology, Danvers, MA, U.S.A.), rabbit anti-LC3 polyclonal antibody (1:1000; Novus Biologicals, Littleton, CO, U.S.A.), rabbit anti-p62 antibody (1:1000; Cell Signaling Technology, Danvers, MA, U.S.A.), rabbit antiadenosine 5′-monophosphate (AMP)-activated protein kinase (AMPK) antibody (1:1000, Cell Signaling Technology, Danvers, MA, U.S.A.), rabbit antiphosphorylated AMPK antibody (1:1000, Cell Signaling Technology, Danvers, MA, U.S.A.) antimammalian target of rapamycin rabbit (mTOR) antibody (1:1000, Cell Signaling Technology, Danvers, MA, U.S.A.), rabbit antiphosphorylated mTOR antibody (1:1000, Cell Signaling Technology, Danvers, MA, U.S.A.), rabbit anti-p70 ribosomal protein S6 kinase (p70S6K) antibody (1:1000, Cell Signaling Technology, Danvers, MA, U.S.A.), rabbit antiphosphorylated p70S6K antibody (1:1000, Cell Signaling Technology, Danvers, MA, U.S.A.), rabbit anticleaved caspase-3 antibody (1:1000; Cell Signaling Technology, Danvers, MA, U.S.A.), rabbit anti-Bax antibody (1:1000; Cell Signaling Technology, Danvers, MA, U.S.A.) and mouse anti-β-actin antibody (1:5000, Beyotime Biotechnology, Shanghai, China). Membranes were then incubated with a Donkey anti-rabbit or Donkey anti-mouse secondary antibody (1:10,000, LI-COR Biosciences, Lincoln, NE, U.S.A.) accordingly. Images were obtained and analyzed using the Odyssey infrared imaging system (LI-COR Bioscience, Lincolin, NE, U.S.A.).

### Transmission electron microscopy

Primary ventricular cardiomyocytes were isolated from neonatal WT or CB2R KO mice and cultured at 37°C on glass coverslips overnight, followed by the treatments mentioned above. Cells were harvested and fixed overnight at 4°C in 2.5% glutaraldehyde in 0.1 M PBS, and then post-fixed in 1% buffered osmium tetroxide for 2 h. Specimens were processed in routine procedure and examined under a transmission electron microscope (H-700; Hitachi, Tokyo, Japan).

### Statistical analysis

All data are presented as mean value ± S.E.M. Statistical analysis was conducted using Student’s *t* test or one-way analysis of variance (ANOVA) followed by Bonferroni *post hoc* test for the comparison of two independent groups or multiple comparisons, respectively. *P*<0.05 was considered as statistical difference between groups.

## Results

### Deletion of CB2R plays a detrimental role in the severity of MI and cardiac function *in vivo*

**Figure 1:**
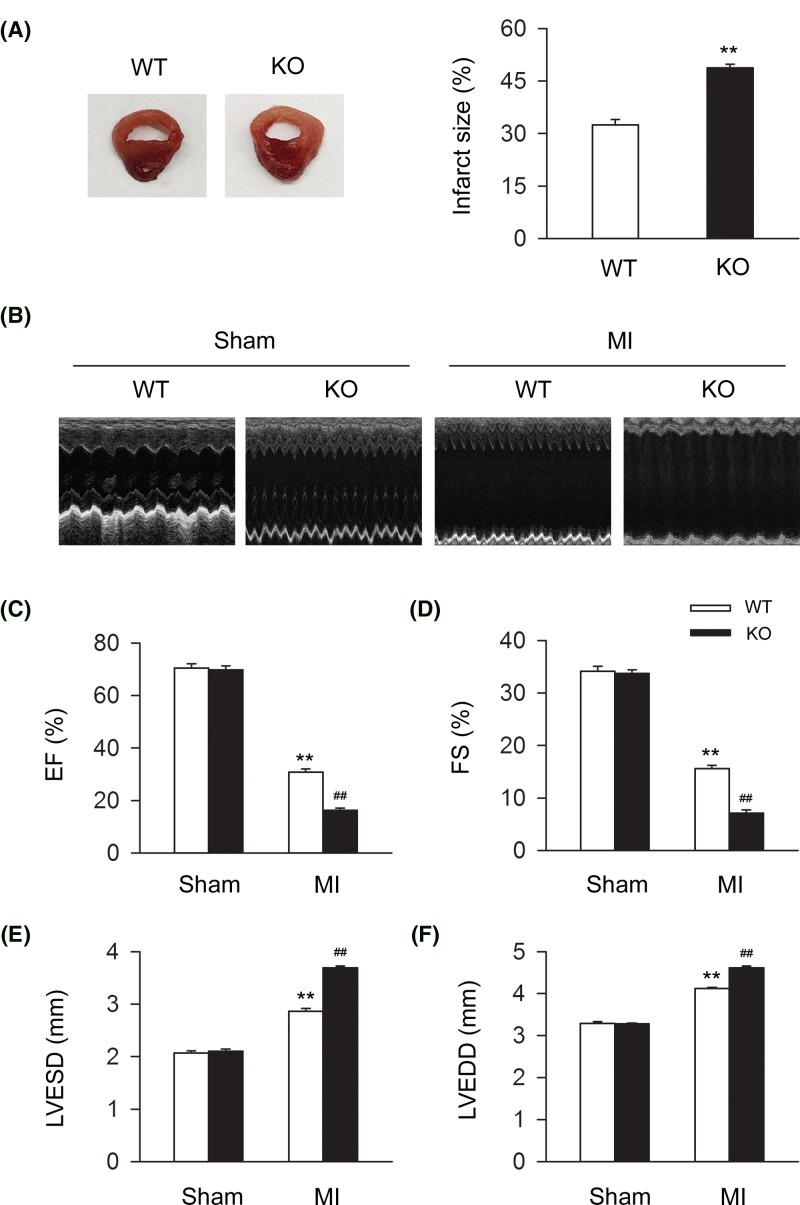
CB2R deficiency deteriorates MI severity and cardiac function in MI mice Left anterior descending coronary artery was permanently occluded to create MI mice model. (**A**) Representative images of MI heart from WT and CB2R KO mice isolated at 24-h postinfarction. Quantitative analysis of the percentage of infarct size in heart issue (*n*=nine per group). **P<0.01 versus WT. (**B**) Representative images of 2D guided M-mode echocardiography at 4-week postinfarction. (**C–F**) Quantitative analysis of 2D guided M-mode echocardiographic detection including EF (%), FS (%), LVESD, and LVEDD at 4-week postinfarction (*n*=seven per group). **P<0.01 versus WT sham; ##*P*<0.01 versus WT MI.

To assess the effect of CB2R on the severity of MI, we detected the ratio of infarct size in WT and CB2R KO MI mice. We found that knocking out CB2R significantly led to the enhancement of infarct size ratio (Figure 1A). To further determine the role of CB2R in cardiac function, we conducted the echocardiographic examination and assessed the cardiac function accordingly in WT and CB2R sham or MI mice. We found that knocking out CB2R significantly led to the deterioration of cardiac function including EF (%), FS (%), LVESD, and LVEDD (Figure 1B–F). Taken together, those data demonstrated the detrimental effect of CB2R deletion in the severity of MI and cardiac function in MI mice.

### Deletion of CB2R leads to a detrimental effect on cardiomyocytes *in vitro*

**Figure 2:**
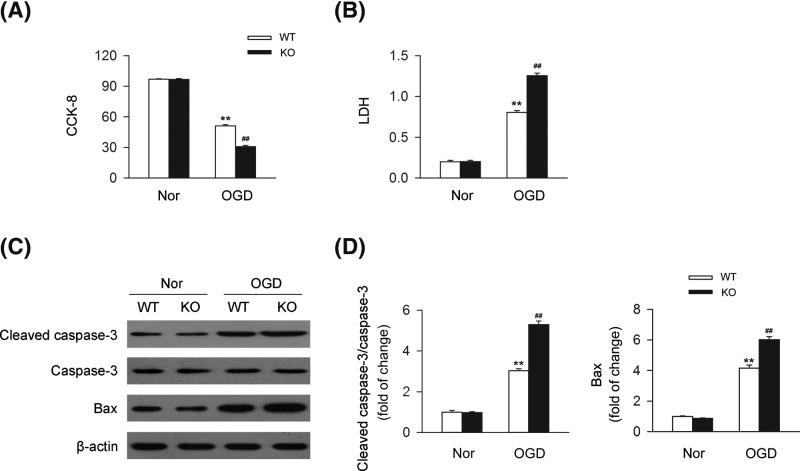
CB2R deficiency leads to the damage of cardiomyocytes under OGD Primary cardiomyocytes isolated from WT or CB2R KO mice were exposed to OGD for 6 h. (**A**) Quantitative analysis of cell viability (CCK-8) in primary cardiomyocytes (*n*=six per group). **P<0.01 versus WT nor; ##*P*<0.01 versus WT OGD. (**B**) Quantitative analysis of LDH release in primary cardiomyocytes (*n*=six per group). **P<0.01 versus WT nor; ##*P*<0.01 versus WT OGD. (**C**) Levels of apoptosis-related proteins including cleaved caspase-3, caspase-3, and Bax were detected by western blot. (**D**) Quantitative analysis of the relative cleaved caspase-3/caspase-3 ratio and Bax expression (*n*=five per group). **P<0.01 versus WT nor; ##*P*<0.01 versus WT OGD. Nor, normal.

To determine the effect of CB2R on cardiomyocytes, we isolated cardiomyocytes from WT or CB2R KO mice and detected the cell viability under OGD or not through CCK-8 and LDH release assay, respectively. We found that knocking out CB2R resulted in the decrease of cell viability and increase of LDH release in cardiomyocytes under OGD (Figure 2A,B). In addition, knocking out CB2R led to the enhancement of the levels of apoptosis-related cleaved caspase-3/caspase-3 ratio and Bax in cardiomyocytes under the challenge of OGD (Figure 2C,D). Those data suggested the detrimental effect of CB2R deletion on cardiomyocytes under OGD.

### Deletion of CB2R deteriorates autophagy process *in vivo*

**Figure 3:**
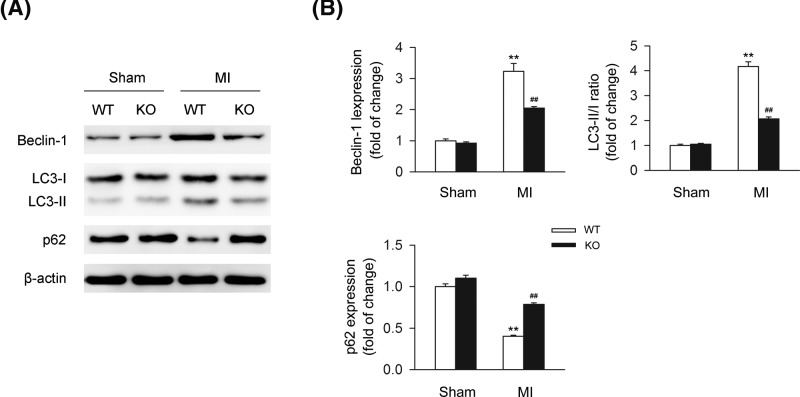
CB2R deficiency results in the dysfunction of autophagy in ischemic heart from MI mice Left anterior descending coronary artery was permanently occluded to create MI mice model. Heart issues were isolated at 24-h postinfarction. (**A**) Levels of autophagy-related proteins including Beclin-1, LC3-II/I, and p62 were detected by western blot. (**B**) Quantitative analysis of the relative Beclin-1, LC3-II/I ratio, and p62 expression (*n*=five per group). **P<0.01 versus WT sham; ##*P*<0.01 versus WT MI.

It was reported previously that cardiac autophagy produced a cardio-protective effect in MI [31]. To determine the association of CB2R and autophagy in MI, we detected the levels of autophagy-related proteins including Beclin-1, LC3-II/I, and p62. We found that under MI, knocking out CB2R largely reduced the levels of Beclin-1 and LC3-II/I ratio, while led to the increase of the level of p62, indicating the positive effect of CB2R on autophagy in MI (Figure 3A,B).

### Deletion of CB2R deteriorates autophagy process of cardiomyocytes *in vitro*

**Figure 4:**
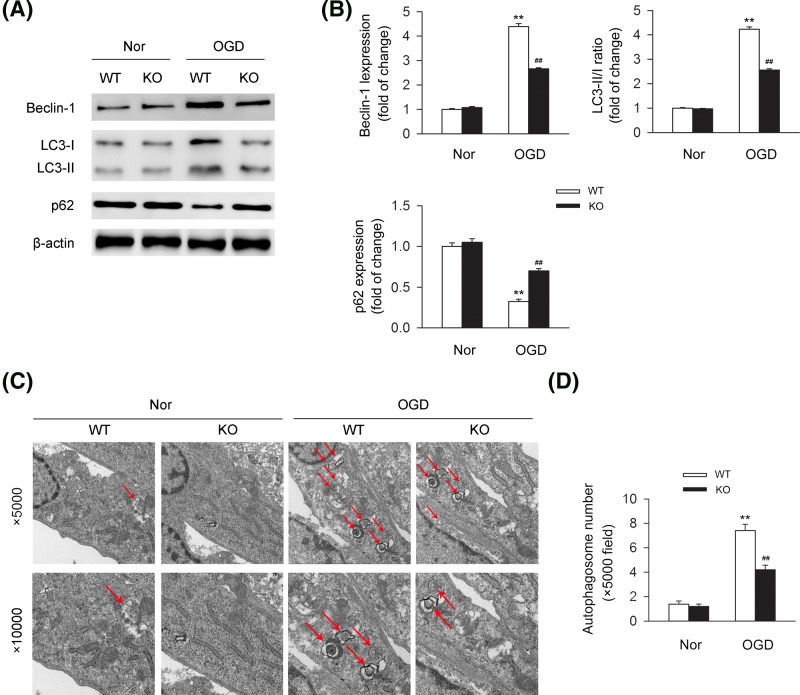
CB2R deficiency results in the dysfunction of autophagy in cardiomyocytes under OGD Primary cardiomyocytes isolated from WT or CB2R KO mice were exposed to OGD for 6 h. (**A**) Levels of autophagy-related proteins including Beclin-1, LC3-II/I, and p62 were detected by western blot. (**B**) Quantitative analysis of the relative Beclin-1, LC3-II/I ratio, and p62 expression (*n*=five per group). **P<0.01 versus WT nor; ##*P*<0.01 versus WT OGD. (**C**) The number of autophagosomes was detected by transmission electron microscopy with lower magnification (×5000, upper line) and higher magnification (×10,000, lower line). (**D**) Quantitative analysis of the number of autophagosomes with lower magnification (×5000) (*n*=five per group). **P<0.01 versus WT nor; ##*P*<0.01 versus WT OGD. Nor, normal.

We further assessed the association of CB2R and autophagy in cardiomyocytes under OGD. Levels of autophagy-related proteins including Beclin-1, LC3-II/I, and p62 were detected in cardiomyocytes under OGD or not. We found that knocking out CB2R largely reduced the levels of Beclin-1 and LC3-II/I ratio under OGD, while led to the increase of the level of p62, indicating the positive effect of CB2R on autophagy in cardiomyocytes *in vitro* (Figure 4A,B). In addition, TEM was applied for the detection of the number of autophagosomes. We found that knocking out CB2R significantly led to the decrease of the number of autophagosomes in cardiomyoctyes under the challenge of OGD (Figure 4C,D).

### CB2R deficiency plays an attenuative role in the cardiac protective effect of rapamycin *in vivo*

**Figure 5:**
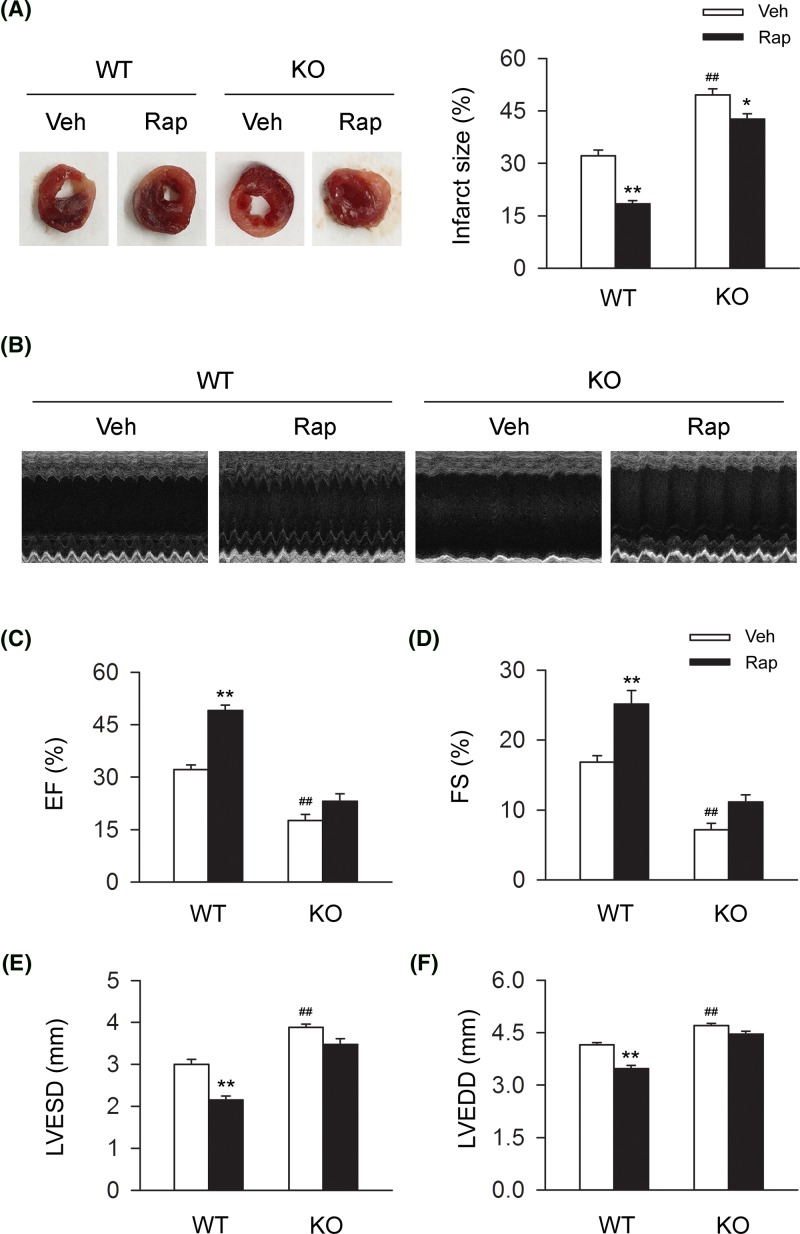
CB2R deficiency largely attenuates the cardiac protective effects of rapamycin in MI mice Left anterior descending coronary artery was permanently occluded to create MI mice model. In two subgroups of MI mice, rapamycin was given daily (2 mg/kg body weight, i.p.) from 1-week preinfarction to occluding surgery. (**A**) Representative images of MI heart from WT and CB2R KO mice isolated at 24-h postinfarction. Quantitative analysis of the percentage of infarct size in heart issue (*n*=nine per group). *P<0.05 versus the corresponding Veh group, **P<0.01 versus the corresponding Veh group; ##*P*<0.01 versus WT Veh group. (**B**) Mice continued to be treated with rapamycin daily (2 mg/kg body weight, i.p.) from 24 h after occluding surgery to 24 h before echocardiographic detection. Representative images of 2D guided M-mode echocardiography at 4-week postinfarction. (**C–F**) Quantitative analysis of 2D guided M-mode echocardiographic detection including EF, FS, LVESD, and LVEDD at 4-week postinfarction (*n*=seven per group). **P<0.01 versus the corresponding Veh group; ##*P*<0.01 versus WT Veh group. Rapa, rapamycin; Veh, vehicle;.

To further determine whether the detrimental effect of CB2R deletion on MI resulted in the deficiency of autophagy, we used rapamycin to induce autophagy in WT and CB2R KO MI mice. We found that the administration of rapamycin significantly reduced the ratio of infarct size in heart issue from WT MI mice, while the alleviative effect of rapamycin was largely attenuated in CB2R KO MI mice (Figure 5A). We further conducted the echocardiographic examination and assessed the cardiac function accordingly in WT and CB2R sham or MI mice. We found that the administration of rapamycin significantly improved the cardiac function including EF (%), FS (%), LVESD, and LVEDD in WT MI mice, while those effects of rapamycin was largely attenuated in CB2R KO MI mice (Figure 5B-F). Taken together, those data demonstrated that CB2R deletion largely attenuated the cardiac protective effect of rapamycin in MI mice.

### CB2R deficiency plays an attenuative role in the cardiac protective effects of rapamycin in cardiomyocytes *in vitro*

**Figure 6:**
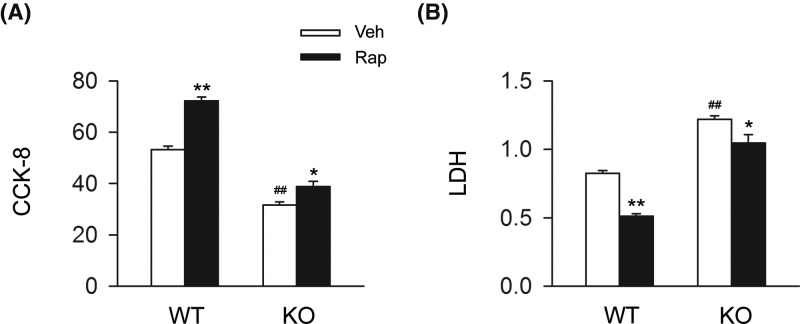
CB2R deficiency largely attenuates the cardiac protective effects of rapamycin in cardiomyocytes under OGD Primary cardiomyocytes isolated from WT or CB2R KO mice were exposed to OGD for 6 h. Rapamycin was pretreated (1 μg/l) at 10 min before exposed to OGD. (**A**) Quantitative analysis of cell viability (CCK-8) in primary myocardiocytes (*n*=six per group). *P<0.05 versus the corresponding Veh group, **P<0.01 versus the corresponding Veh group; ##*P*<0.01 versus WT Veh group. (**B**) Quantitative analysis of LDH release in primary myocardiocytes (*n*=six per group). *P<0.05 versus the corresponding Veh group, **P<0.01 versus the corresponding Veh group; ##*P*<0.01 versus WT Veh group.

We then determined whether the detrimental effect of CB2R deletion on cardiomyocytes under OGD resulted in the deficiency of autophagy. We found that the administration of rapamycin significantly increased cell viability and decreased LDH release in cardiomyocytes from WT mice exposed to OGD, while the cardiac protective effect of rapamycin was largely attenuated in cardiomyocytes from CB2R KO mice exposed to OGD (Figure 6A,B). Those data indicated that CB2R deletion largely attenuated the cardiac protective effect of rapamycin in cardiomyocytes under OGD.

### CB2R-induced autophagy is mediated by AMPK-mTOR-p70S6K signaling pathway

**Figure 7:**
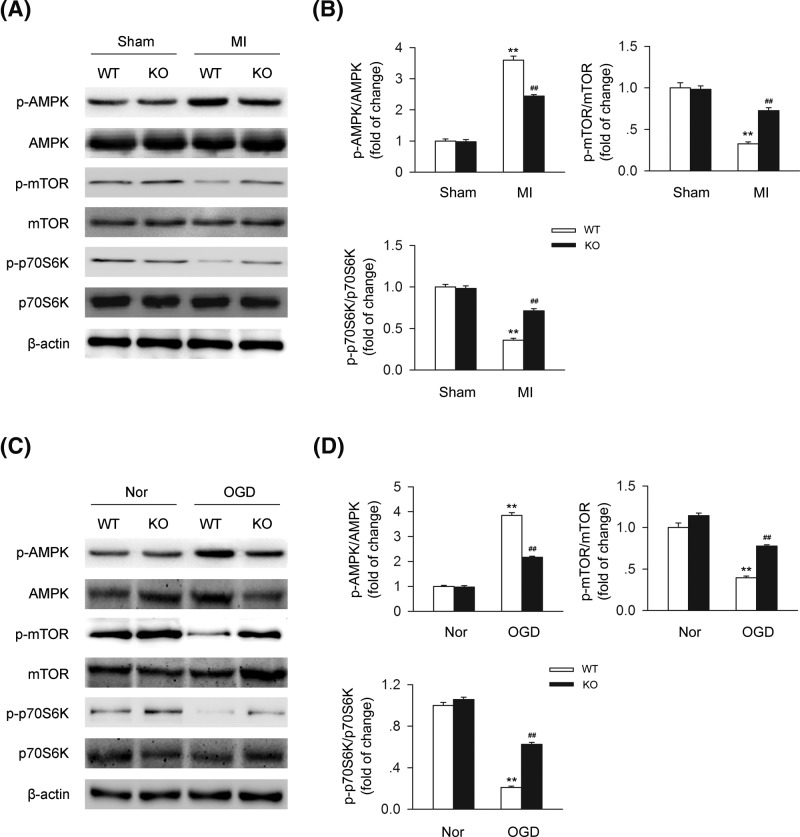
AMPK-mTOR-p70S6K signaling pathway is involved in the process of CB2R-mediated autophagy (**A**) Left anterior descending coronary artery was permanently occluded to create MI mice model. Heart issues were isolated at 24-h postinfarction. Levels of AMPK-mTOR-p70S6K signaling-related proteins were detected by western blot. (**B**) Quantitative analysis of the relative p-AMPK/AMPK, p-mTOR/mTOR, and p-p70S6K/p70S6K ratio (*n*=five per group). **P<0.01 versus WT sham; ##*P*<0.01 versus WT MI. (**C**) Primary cardiomyocytes isolated from WT or CB2R KO mice were exposed to OGD for 6 h. Levels of AMPK-mTOR-p70S6K signaling-related proteins were detected by western blot. (**D**) Quantitative analysis of the relative p-AMPK/AMPK, p-mTOR/mTOR, and p-p70S6K/p70S6K ratio (*n*=five per group). **P<0.01 versus WT nor; ##*P*<0.01 versus WT OGD. Nor, normal.

AMPK-mTOR-p70S6K signaling pathway was regarded as a classic inductive pathway of autophagy, involved in the regulation of various kinds of diseases. Here we demonstrated whether this pathway was involved in this process. We detected the levels of AMPK-mTOR-p70S6K pathway-related proteins in heart issues isolated from WT or CB2R KO sham or MI mice and found that knocking out CB2R was significantly suppress AMPK-mTOR-p70S6K signaling pathway in MI (Figure 7A,B). We further detected the levels of AMPK-mTOR-p70S6K pathway-related proteins in cardiomyocytes from WT or CB2R KO mice under OGD and found that knocking out CB2R was significantly suppress AMPK-mTOR-p70S6K signaling pathway under OGD (Figure 7C,D). Those data indicated that CB2R-induced autophagy is mediated by AMPK-mTOR-p70S6K signaling pathway both *in vivo* and *in vitro*.

## Discussion

CB2R has been reported to play an attenuative role in the pathogenesis and progression of MI [19,21,32, 33]. For instance, it was previously demonstrated that activating CB2R functioned in the decrease of infarct area and adverse myocardial remodeling through the down-regulation of inflammatory response [32]. In addition, Maslow et al. showed that cannabinoids could produce an infarct-reducing effect of early ischemic preconditioning, delayed ischemic preconditioning and ischemic postconditioning against myocardial ischemia/reperfusion [19]. They also reported that peripheral activation of CB2R by HU-210 could inhibit the Na^+^/Ca^2+^ exchange, thus producing an anti-apoptotic and cardiac protective effect [19]. Besides, it was reported that HU-308, a selective CB2R agonist, significantly reduced the infarct size and levels of reactive oxygen species and tumor necrosis factor-α in MI or ischemia-reperfusion mice model, thus protecting against cardiac injury [21]. Consistent with those studies, in the present study, we found that knocking out CB2R (CB2R KO) largely increased the ratio of infarct size and damaged the cardiac function index including EF (%), FS (%), LVESD, and LVEDD in MI mice. We further demonstrated the detrimental effects of CB2R deletion on cell viability in cardiomyocytes in *in vitro* studies. Collectively, those data indicated that CB2R played an important role in the protection of cardiomyocytes under the disease of MI.

As mentioned previously, autophagy has been regarded as a vital catabolic process in organisms. So far, accordingly to the latest guidelines for monitoring autophagy, various kinds of techniques are available for the analysis of autophagy [34]. Autophagy is involved in the regulation of various kinds of disorders. In MI, it was previously reported that inducing autophagy process by salvianolic acid B produced a cardiac protective effect in acute MI model through the promotion of neovascularization and inhibition of apoptosis [33]. In addition, it was also reported by Zhang et al. that berberine could play an attenuative role in adverse left ventricular remodeling and cardiac dysfunction after MI through enhancing the level of autophagy process in cardiomyocytes [35]. Consistently, data from our current study demonstrated that the administration of rapamycin could largely reduced the infarct size of heart issues from MI mice as well as improvement of cell viability in cardiomyocytes under OGD, thus indicating the anti-infarcted and cardiac protected effects of inducing autophagy process.

For the relations between CB2R and autophagy, it was previous demonstrated by Shao et al. that selective activation of CB2R with HU-308 significantly enhanced the level of autophagy process in experimental autoimmune encephalomyelitis through the suppression of inflammatory reaction, thus protecting against the disease [36]. However, deletion of CB2R led to the reverse effects [36]. Similar results were reported by them in inflammatory bowel disease, further indicating the inductive role of activating CB2R in autophagy and its anti-inflammatory effects [37]. In addition, it was demonstrated that selective activation of CB2R by JMH-133 suppressed the inflammatory reaction and steatosis in murine alcohol-induced liver through an autophagy-dependent pathway, suggesting the inductive role of activating CB2R in autophagy process [38]. Here in our current study, we explored the relations between CB2R and autophagy in MI. We found that the level of autophagy was significantly decreased in the infarcted heart issues from CB2R KO MI mice compared with the WT MI mice. Similar trends of change on the level of autophagy was detected in cardiomyocytes with the exposure of OGD. Those data indicated that CB2R could induce autophagy process in cardiomyocytes under ischemic stress. Subsequent experiments uncovered that deletion of CB2R could attenuate the alleviative effect of inducing autophagy through rapamycin in MI mice, suggesting that CB2R could protect against MI through the maintaining of autophagy process. Similar data were demonstrated in cardiomyocytes with the exposure of OGD.

It has been widely reported by previous studies that apoptosis contributes greatly to the pathogenesis and progression of MI [39–41]. For example, Santos-Gallego et al. demonstrated that the application of Fingolimod produced a cardioprotective effect against myocardial ischemia-reperfusion injury through the inhibition of apoptosis [39]. Here, in our current study, we showed that apart of inducing autophagy, CB2R deletion significantly enhanced cardiac apoptosis via increasing apoptosis-related cleaved caspase-3/caspase-3 ratio and Bax in cardiomyocytes under the challenge of OGD. To clarified the effect of CB2R deletion in MI mice models *in vivo* as well as exploring specific mechanisms related to apoptosis, further studies are demanded on this issue.

We finally explored the signaling pathway involving this process. We found that AMPK-mTOR-p70S6K signaling pathway, the classic autophagy-related pathway, participated in the medication of the CB2R-induced autophagy in *in vivo* and *in vitro* studies. It was previously demonstrated that activating CB2R could induce the AMPK-mTOR-p70S6K signaling-mediated autophagy in macrophages, thus ameliorating the DSS-induced colitis mice model [37]. In ischemca/reperfusion injury and diabetic cardiomyopathy, AMPK-mTOR-p70S6K signaling was detected to induce the protective autophagy by ischemic postconditioning, indicating the involvement of AMPK-mTOR-p70S6K signaling-mediated autophagy [42,43].

Taken together, here in the current study, we demonstrated for the first time that CB2R played an ameliorated role in the pathogenesis and progression of MI through the AMPK-mTOR-p70S6K signaling-mediated autophagy. We found that deletion of CB2R led to a detrimental effect in MI mice models and cardiomyocytes under OGD as well as a decrease of autophagy level. In addition, knocking out CB2R attenuated the cardiac protective effects led to by activating autophagy. We believe that the present study might provided a novel strategy in the treatment of MI in the future.

## Abbreviations

AMP: adenosine monophosphate
AMPK: AMP-activated protein kinase
CB2R: cannabinoid receptor 2
EF: ejection fraction
FS: fractional shortening
KO: knockout
LDH: lactate dehydrogenase
LVESD: ventricular end-systolic diameter
LVEDD: left ventricular end-diastolic diameter
MI: myocardial infarction
mTOR: mammalian target of rapamycin rabbit
OGD: oxygen-glucose deprivation
WT: Wild type
